# A new freshwater snail (Gastropoda, Pomatiopsidae) endemic to Fuxian Lake (Yunnan, China) identified, based on morphological and DNA evidence

**DOI:** 10.3897/BDJ.8.e57218

**Published:** 2020-11-03

**Authors:** Ling Shi, Yu Shu, Chen Qiang, Ping Xu, Ying Tian, Yaqing Chang

**Affiliations:** 1 Key Laboratory of Mariculture & Stock Enhancement in North China Sea, Ministry of Agriculture, Dalian Ocean University, Dalian, China Key Laboratory of Mariculture & Stock Enhancement in North China Sea, Ministry of Agriculture, Dalian Ocean University Dalian China; 2 Dalian Shell Museum, Dalian, China Dalian Shell Museum Dalian China

**Keywords:** taxonomy, Yunnan, *
Lacunopsis
*, 18S rDNA, freshwater gastropod

## Abstract

**Background:**

*Lacunopsis* Deshayes, 1876 is restricted to South Asia and shows a remarkable regional distribution. Fifteen species have been reported from the lower Mekong River area of Laos, Cambodia, Thailand and Vietnam. Two species, *Lacunopsis auris* Y.-Y. Liu, Y.-X. Wang & W.-Z. Zhang, 1980 and *L. yunnanensis* Y.-Y. Liu, Y.-X. Wang & W.-Z. Zhang, 1980 occur in the Yunnan Province of China. The most recent treatments of *Lacunopsis* date back to the 1970s and 1980s, therefore detailed information on anatomy and DNA analysis is lacking.

**New information:**

A new species of freshwater snail (Gastropoda, Pomatiopsidae), *L. yuxiensis* sp. nov., is described, based on samples collected from Fuxian Lake (Yunnan, China). The new species is characterised by a solid and dark red shell with yellow spiral ribs on the whorls, reminiscent of marine gastropods of the family Trochidae. In addition to a description of the shell morphology and radula, molecular data are presented for the first time. This compilation of faunal and taxonomic data provides relevant information for use in conservation efforts. Additional molecular data may prove helpful for both reﬁning current knowledge on the morphological variability within this species and verifying the degree of cryptic diversity of this genus.

## Introduction

The Yunnan Plateau of south-western China has an elevation of approximately 2000 m above sea level and contains many lakes (Ley et al. 1963). These lakes harbour a unique fauna of at least 124 gastropod species, most of which are endemic to the region (Zhang and Qi 1957, Sun 1959, Zhang et al. 1997). However, research on gastropods in Yunnan is currently limited; the endemic gastropod species in many lakes have yet to be described and modern phylogenetic studies on gastropods are scarce (Zhang et al. 2015).

Speciation of aquatic fauna is obvious in the isolated plateau lakes in Yunnan, which have many endemic species (Wang and Wang 2010). However, the development of industry and agriculture in the surrounding areas of the lakes has caused a series of environmental problems, resulting in decreased biodiversity (Ding et al. 2017).

During a survey conducted in October 2019 of the freshwater gastropod fauna in Yunnan, a new species of gastropod belonging to *Lacunopsis* Deshayes, 1876 was found in the shallow waters of the nearby Fuxian Lake. Here, we describe the new species and provide faunal information on aquatic system gastropod diversity and endemism with relevance to conservation efforts.

The family Pomatiopsidae comprises 36 genera distributed across Asia, South America, North America, Africa and Australia. The peculiar biogeographical patterns of pomatiopsids across the planet, that is, the occurrence of endemic genera on the southern continents and a high biodiversity in southeast Asian river systems, have raised questions regarding the Gondwanaland origin of the Pomatiopsidae (Lydeard and Cummings 2019). There are close to 170 existing names available for extant Pomatiopsidae species (Strong et al. 2008). Pomatiopsidae is a family of the Caenogastropod clade of Gastropoda. Pomatiopsids are characterised by a sessile habit and uncoiled shells and their taxonomy has remained in a state of ﬂux for decades owing to highly-variable shell features (Lydeard and Cummings 2019). Most species descriptions are limited to shell features and there is a general lack of information on the soft parts and other features of living Pomatiopsids.

The genus was described by Deshayes (1876) within the literature work of Deshayes and Jullien (1876) and contained fifteen species before the current study (Bavay 1896, Brandt 1974, Davis 1979, Deshayes and Jullien 1876, Poirier 1881, Liu et al. 1980). The highest biodiversity of *Lacunopsis* is found in the lower Mekong River area of Laos, Cambodia, Thailand and Vietnam (Lydeard and Cummings 2019). Two species are distributed over Yunnan Province in China (Liu et al. 1980); however, they lack detailed radula and DNA descriptions.

Here, to reﬁne our knowledge of the morphological variability within a *Lacunopsis* species and to verify the degree of cryptic diversity in this genus, we provided SEM radula images of *Lacunopsis* and 18S rDNA information as well.

## Materials and methods

### Geographic information on locality

**Figure 1.**
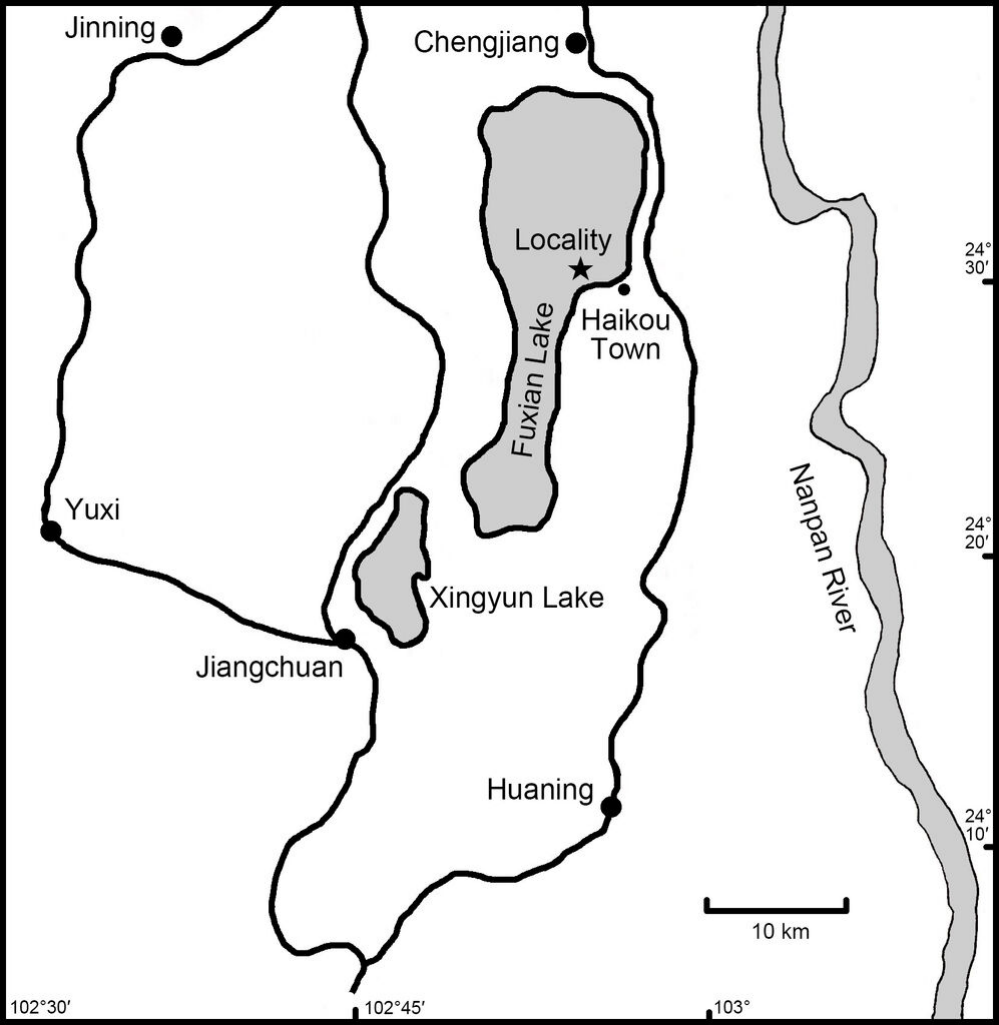
Sampling location in Fuxian Lake.

Fuxian Lake, located in Yunnan Province, is the second-deepest plateau freshwater lake in China (Fig. 1). The lake has a total area of approximately 212 km^2^ and an elevation of 1721 m above sea level, with maximum and average depths of 155 m and 89.7 m, respectively. Fuxian Lake and Xingyun Lake are separated by a mountain and linked by a river (He et al. 2019, Xiong and Li 2008).

### Sample collection

Specimens were collected by diving and hand netting (depths of 1–6 m). Morphometric variables of shells were measured using a Vernier caliper (SYNTEK:SY168-150). The shells were photographed with a digital camera (Leica DM750). Living specimens were extracted from their shells, photographed with a digital camera attached to a stereomicroscope (CEWEI PXS9-T) and then ﬁxed in 95% ethanol and 4% formalin for molecular analysis and anatomical study, respectively. Radula and operculum were dissected from the buccal mass, soaked in 10% sodium hydroxide (NaOH) solution and rinsed with distilled water. Whenever it was necessary to remove debris and encrustations, for example, from operculum, an ultrasonic water bath was used. For scanning electron microscopy (SEM) observations, the radula was dehydrated in progressive concentrations of ethanol, the operculum and shell were thoroughly dried after use of the ultrasonic water bath, mounted on stubs and gold sputtered. The SEM analysis was performed at a working distance of 5–15 mm and at 10.0 kilovolts using a tabletop SEM (TESCAN rega3) at the Key Laboratory of Mariculture & Stock Enhancement in North China's Sea, Ministry of Agriculture and Rural Affairs, China.

As the preservation medium often made it difficult to remove tissues, the shells were, therefore, by necessity, broken into pieces using a hammer before SEM observation. Thus, the shells could not be completely preserved.

### Radula preparation

Shells were broken into pieces using a hammer and the whole visceral mass was soaked in 10% NaOH for 24 h to dissolve the soft tissues. Following this, the remaining muscular and connective tissues were stripped from the radula using tweezers and a dissecting needle under the same stereomicroscope. Each radula was washed with 10% sodium hypochlorite solution for several minutes to completely dissolve the remaining soft tissues. The radula was then cleaned with an ultrasonic cleaner (Power 50 W) for 2 min, rinsed with distilled water, dried, mounted on stubs and used for the next step or stored for no longer than two weeks. The radula was photographed using the same tabletop SEM as described above. Specimens were mounted on sticky tabs, gold sputter-coated and imaged under low vacuum.

### DNA extraction

Total genomic DNA was isolated from a small piece of tissue taken from the foot of each ethanol-preserved specimen (n = 20). Extraction was performed using the Ezup Column Animal Genomic DNA Purification Kit (Sangon Biotech Co. Ltd. Shanghai, China) in the Key Laboratory of Mariculture & Stock Enhancement in North China’s Sea, Ministry of Agriculture and Rural Affairs, China. All extracted DNA was stored at 4°C for short-term use. Undiluted or variable dilutions (ranging from 1:10 to 1:50, based on the DNA concentration) of each extracted DNA were used as template DNA for PCR amplification of a portion. For our target loci 18S rDNA, the primers used were 18S-F 5′-AACCTGGTTGATCCTGCCAGT-3′ and 18S-R 5′-TGATCCTTCTGCAGGTTCA-3′ (Medlin et al. 1988). The PCR conditions were as follows: initial denaturation at 95°C for 5 min; 35 cycles of denaturation at 95°C for 45 s, annealing at 45°C for 45 s and extension at 72°C for 1 min and 30 s; and final extension at 72°C for 5 min. Each PCR reaction was performed in a total volume of 25 µl, including 2.5 µl of 2 mM of each dNTP (Sangon Biotech Co. Ltd.), 2.5 µl of 10× loading buffer-MgCl_2_ (Qiagen), 2.5 µl of 2 µM of each primer, 0.25 µl of 5 U/µl Taq DNA polymerase (Qiagen), 19 µl of demineralised water and 1 µl of the DNA template. Amplified products were purified using the SanPrep Column PCR Product Purification Kit (Sangon Biotech Co. Ltd. Shanghai, China) and preserved at -20°C for sequencing. Sequencing was done by Sangon Biotech Co. Ltd. (Shanghai, China) and what we ended up with was a SEQ file containing an 18S rDNA fragment.

## Taxon treatments

### Lacunopsis yuxiensis sp. n.

B6662182-ECC6-5EEE-8291-A45C4E6EA214

14633802-E974-4B36-8CC7-D6DD45B34C60

#### Materials

- **Type status:** Holotype. **Occurrence:** catalogNumber: DLSM0001; **Taxon:** scientificNameID: urn:lsid:zoobank.org:pub:C1FF9D49-158C-4D86-A3D4-8E8818CC2DD8; phylum: Mollusca; class: Gastropoda; order: Littorinimorpha; family: Pomatiopsidae; genus: Lacunopsis; infraspecificEpithet: yuxiensis; taxonRank: species; **Location:** country: China; stateProvince: Yunnan Province; municipality: Yuxi; locality: Fuxian Lake; verbatimDepth: 10 m; locationRemarks: alive on stones hiding under the grass, at depth of 1–6 m; verbatimLatitude: 24°18'14.53''N; verbatimLongitude: 102°46'42.76''E; **Event:** eventDate: 04-2019; **Record Level:** institutionCode: DLSM; basisOfRecord: PreservedSpecimen

#### Description

**Figure 2.**
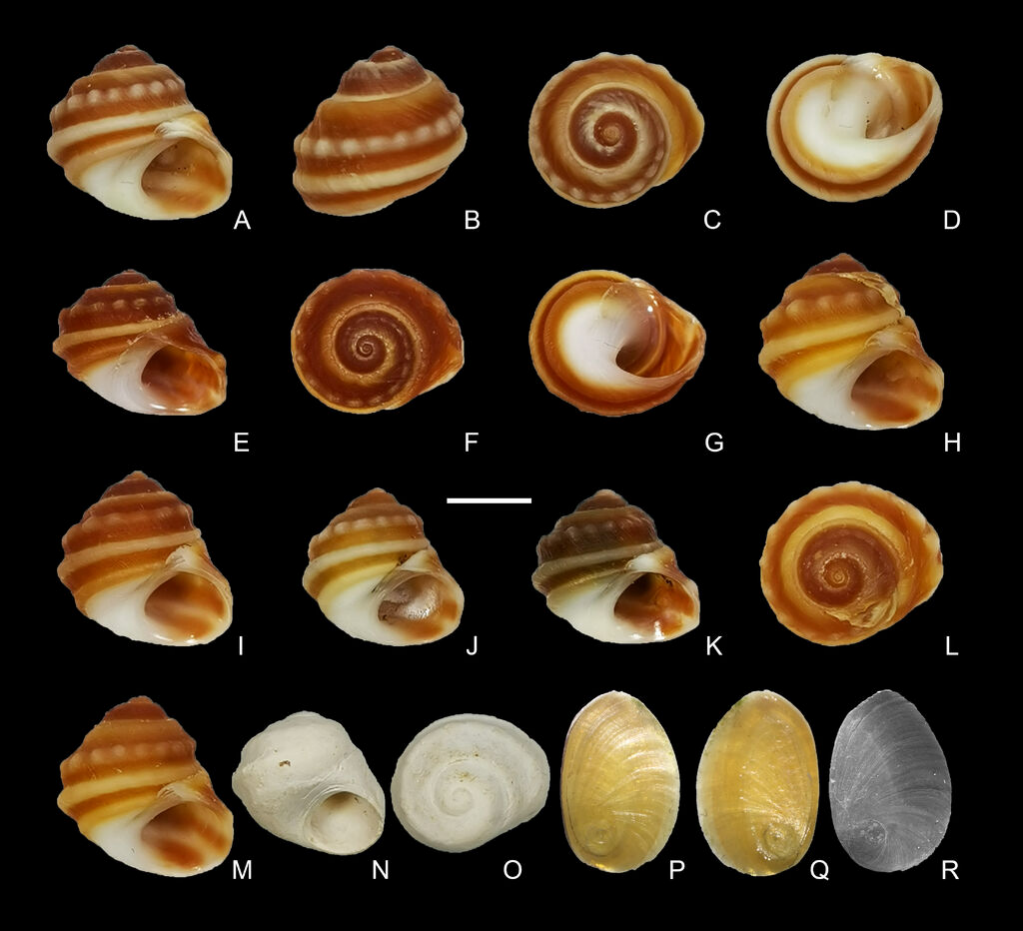
*Lacunopsis yuxiensis* sp. nov. shells. **A–D.** holotype; **E–G.** paratype 1; **H, L.** paratype 2; **I.** paratype 3; **J.** paratype 4; **K.** paratype 5; **M.** paratype 6; **N–O.** subfossil; **P–Q.** operculum; **R.** SEM of the operculum. Scale bar = 5 mm (A–O); operculum size is 6 mm × 4 mm (P–R).

**Figure 3.**
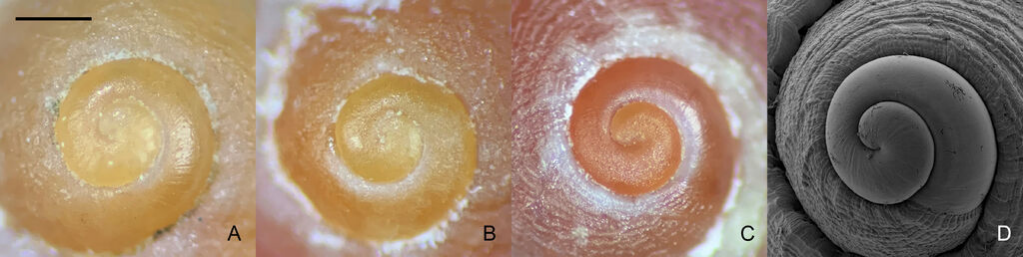
*Lacunopsis yuxiensis* sp. nov. protoconch. **A, B.** yellowish-orange protoconch; **C.** Orange red protoconch; **D.** SEM of the protoconch. Scale bar = 500 µm.

**Shell**: Shell (Fig. 2) small, thick, solid, with low conical spire. Apex obtuse. Four to five whorls, body whorl large, taking up most of the shell. Shell usually dark red (occasionally brown) with four yellow spiral ribs; ribs not obvious in the early whorls. First spiral rib below the suture, wide and usually yellow; second spiral rib granulated; third spiral rib strong; fourth spiral rib on base of the shell, gracile and not obvious. Umbilicus absent, base white, aperture round, slightly concave at sides, operculum smaller than aperture, corneous, thin and ovate, yellow and translucent, length 6 mm, width 4 mm (Fig. 2P–R). Lip thickened, inner lip thick and smooth, without columellar folds. Protoconch yellowish-orange or orange red (Fig. 3), originally smooth (Fig. 3A), but usually appears rough owing to corrosion and abrasion (Fig. 3B).

**Figure 4.**
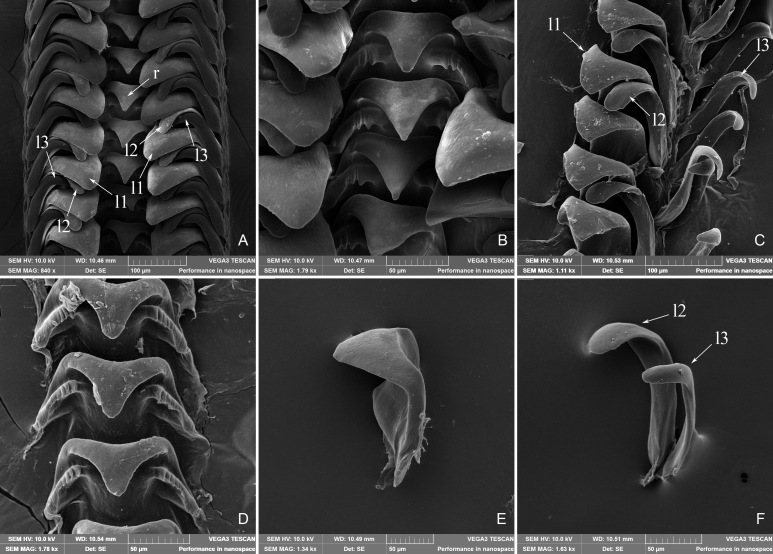
*Lacunopsis yuxiensis* sp. nov. radula. **A.** Front view of radula, r: rachidian tooth, l: lateral teeth; **B.** Magnified view of panel A showing the rachidian tooth; **C.** The separated lateral teeth; **D.** The separated rachidian tooth; **E.** Magnified view of l1; **F.** Magnified view of l2 and l3.

**Radula**: Radula (Fig. 4) length 4 mm, comprising at least 60 rows. Formula 0+3+1+3+0. Rachidian tooth wide, cusp triangular, obtuse and smooth, there are wing-like protrusions on both sides of the shafts and 5–6 rib decorations on the edges of the protrusions. Three lateral teeth (including l1, l2, l3), l1 is closest to the rachidian tooth, cusp triangular, large and wide, more obtuse than the rachidian tooth; l2 and l3 cusp obtuse, more thin and slender. No marginal teeth.

**Figure 5.**
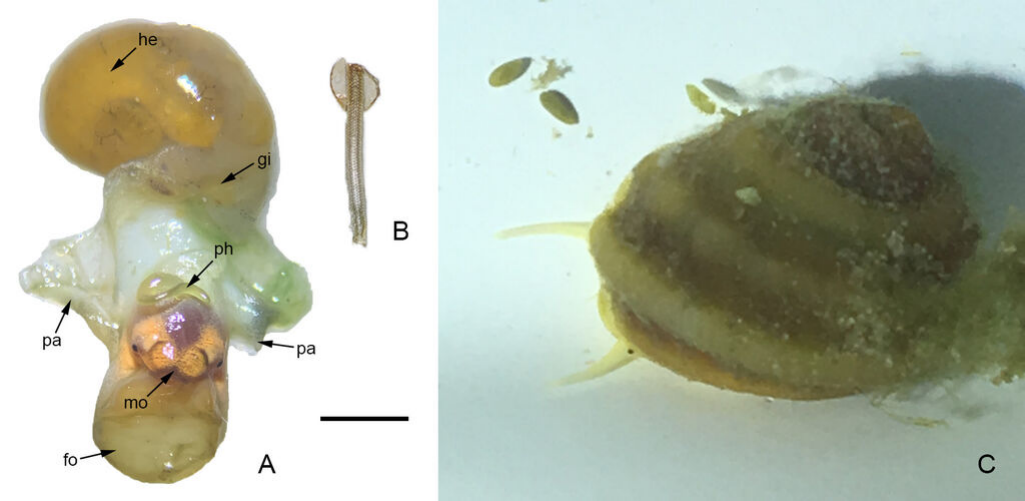
*Lacunopsis yuxiensis* sp. nov. photographs. **A.** dissection with labelled structures; **B.** radula; **C.** photo of live specimen. Scale bar A, B = 2 mm. Abbreviations: fo = foot, gi = gill, he = hepatopancreas, mo = mouth, pa = pallium, ph = phallus.

**Anatomy**: The *Lacunopsis* species has not been anatomically studied previously. In this study, a male specimen (Fig. 5A, B) was dissected.

#### Diagnosis

Shell (Table 1, Fig. 2) small, solid, dark red, comprising four to five whorls, with four yellow spiral ribs and the second one granulated. Lip distinctly thickened, inner lip thick and smooth, without any columellar folds, umbilicus absent, operculum corneous and ovate, radula formula 0+3+1+3+0.

#### Etymology

This species takes the name of its type locality, the city of Yuxi, Yunnan Province.

#### Taxon discussion

**Figure 6.**
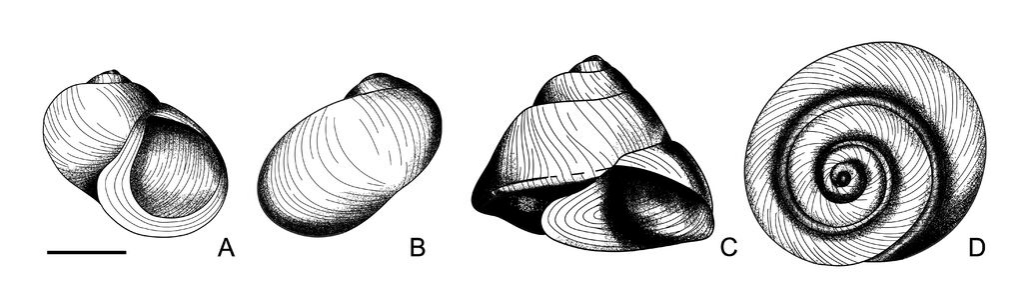
Original figures of two known *Lacunopsis* species (*Lacunopsis auris* and *Lacunopsis yunnanensis*) in Yunnan, China (Liu et al. 1980). **A–B**. *Lacunopsis auris* Y.-Y. Liu, Y.-X. Wang & W.-Z. Zhang, 1980; **C–D.** *Lacunopsis yunnanensis* Y.-Y. Liu, Y.-X. Wang & W.-Z. Zhang, 1980. Scale bar = 1 mm.

The morphology of *L. yuxiensis* sp. nov. is significantly different from that of *L. auris* (Fig. 6A, B) and *L. yunnanensis* (Fig. 6C, D), which are also from Yunnan, China (Liu et al. 1980). Firstly, the shells of *L. auris* and *L. yunnanensis* are very small around 2-3 mm, while the shell of *L. yuxiensis* sp. nov. is around 10 mm. Secondly, *L. yuxiensis* sp. nov. is characterised by its four yellow spiral ribs, while *L. auris* and *L. yunnanensis* is not. The four ribs have different appearances, the first spiral rib is below the suture between the body whorl and penultimate whorl, it is wide and usually yellow; the second spiral rib is granulated; the third spiral rib is strong; and the fourth spiral rib is on the base of the shell, gracile and not obvious (Fig. 2A–M).

#### DNA analysis

**Figure 7.**
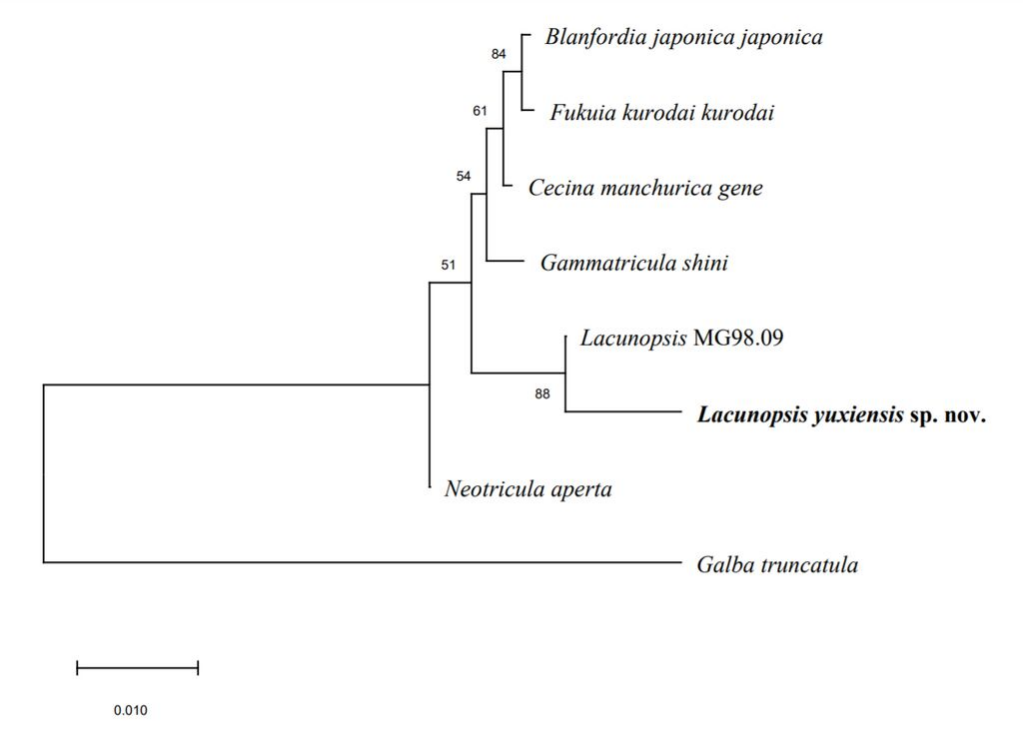
Neighbour-joining phylogenetic tree of *Lacunopsis yuxiensis* sp. nov. within the Pomatiopsidae, using partial 18S rDNA sequences. *Lacunopsis* MG98.09 is the name of this species recorded on the National Center for Biotechnology Information database.

For the phylogenetic analysis of 18S rDNA sequence fragments, we included eight specimens representing eight species (18S gene sequences downloaded from GenBank), with seven species belonging to the family Pomatiopsidae, one species from Lymnaeidae being used as the outgroup. The partial 18S sequence length of *L. yuxiensis* sp. nov. is 987 bp. Accession numbers of the sequences downloaded from GenBank are shown in *Table 2*. All seqeunces were aligned by clustalW in MEGA X (Sudhir et al. 2018). The phylogenetic tree was constructed using MEGA X (Sudhir et al. 2018) by the neighbour-joining method (Fig. 7), with a Kimura 2-parameter substitution model, the numbers at the nodes indicating the bootstrap proportions and 1000 replicates were calculated to obtain bootstrap supports. *L. yuxiensis* sp. nov. and *Lacunopsis* MG98.09 together formed a well-supported clade in the phylogenetic tree and they could be distinguished from the other five freshwater gastropod taxa in the family (Fig. 7). The DNA analysis provides support for the taxonomic placement of the new species.

## Discussion

This new species is placed in *Lacunopsis*, based mainly on DNA analysis and shell morphology. The new species we found herein has the characteristics of shell depressed, solid, spire low, convex and obese, circulated submarginal angles, columellar wide as its original description and the results of 18S rDNA analysis also provide support for the taxonomic placement of the new species.

*Lacunopsis* was first described in 1876, the author describing three species of the genus (*L. Lacunopsis*, *L. monodonta* and *L. tricostatus*), but did not specify which was the type species and some researchers suggested that some of these belonged to *Jullienia*. There are only fuzzy hand-painted drawings of the three species in the original description of *Lacunopsis*, information on the remaining species being also limited. In this regard, Liu et al. (1980) studied the radula and reproductive system of two *Lacunopsis* species from Yunnan and described their morphology. However, her results were presented with rather uninformative hand-drawn sketches. We only found one 18S sequence from *Lacunopsis* from Kameda and Kato (2011), whereas there was no particular information of the shell.

Through the observation of SEM radula images, we found that the teeth cusps were large and obtuse, suggesting a vegetarian feeding habit. The serrated bulges in the shaft of the rachidian tooth are fit for scraping algae off the rocks. In fact, most of the specimens we obtained were wrapped with algae. This feature can also prove the feeding habits of *L. yuxiensis* sp. nov. For the radula, we attempted to spread the radula several times to make the lateral radula clearer, but ultimately failed. The picture shown here is the best from amongst those acquired. The same problem occurred when we performed the dissection. The information available on this genus is scarce and much remains unknown, especially the information regarding anatomy and DNA and requires further systematic research.

## Supplementary Material

XML Treatment for Lacunopsis yuxiensis

## Figures and Tables

**Table 1.** Measurements of *Lacunopsis yuxiensis* sp. nov. (in mm). Abbreviations: H = shell height; W = shell width; AH = height of aperture; AW = width of aperture; BH = height of body whorl.

	Number	H	W	AH	AW	BH
Holotype	DLSM0001	9.84	10.15	4.75	4.58	8.86
Paratypes 1	DLSM0002	9.09	10.08	5.07	4.82	8.32
Paratypes 2	DLSM0003	10.15	10.40	5.70	4.89	9.22
Paratypes 3	DLSM0004	10.64	10.17	5.22	5.05	9.47
Paratypes 4	DLSM0005	9.17	9.23	5.04	4.83	8.50
Paratypes 5	DLSM0006	9.45	9.63	4.96	4.91	8.65
Paratypes 6	DLSM0007	11.12	10.35	4.80	5.06	9.78

**Table 2.** Nucleotide compositions of partial 18S rDNA sequences of specimens investigated in this study.

Family	Genus	Species	GenBank	References
Pomatiopsidae	* Blanfordia *	*Blanfordia japonica japonica*	AB611724.1	Kameda and Kato 2011
	* Cecina *	*Cecina manchurica*	AB611744.1	Kameda and Kato 2011
	* Fukuia *	*Fukuia kurodai kurodai*	AB611764.1	Kameda and Kato 2011
	* Gammatricula *	*Gammatricula shini*	AB611796.1	Kameda and Kato 2011
	* Lacunopsis *	*Lacunopsis* MG98.09	AF212910.1	Wilke et al. 2000
		*Lacunopsis yuxiensis* sp. nov.	MT237175	this research
	* Neotricula *	*Neotricula aperta*	AF531540.1	Attwood et al. 2003
Lymnaeidae	* Galba *	*Galba truncatula*	Y09019.1	Marquez Unpublished
